# Tagged actin mRNA dysregulation in IGF2BP1${}^{-/-}$ mice

**DOI:** 10.1073/pnas.2208465119

**Published:** 2022-09-06

**Authors:** Leti Núñez, Adina R. Buxbaum, Zachary B. Katz, Melissa Lopez-Jones, Chiso Nwokafor, Kevin Czaplinski, Feng Pan, Jason Rosenberg, Hannah R. Monday, Robert H. Singer

**Affiliations:** ^a^Department of Anatomy and Structural Biology, Albert Einstein College of Medicine, New York, NY 10461;; ^b^Regeneron, Tarrytown, NY 10591;; ^c^Genentech, San Francisco, CA 94080;; ^d^National Institutes of Health, Bethesda, MD 20892;; ^e^Eli Lilly and Company, Indianapolis, IN 46285;; ^f^Princeton University, Princeton, NJ 08544;; ^g^University of California, Berkeley, CA 94720;; ^h^Gruss-Lipper Biophotonics Center, Albert Einstein College of Medicine, New York, NY 10461

**Keywords:** RNA binding, mRNA localization, knockout mouse, IGF2BP1

## Abstract

Gene expression is tightly regulated by RNA-binding proteins (RBPs) to facilitate cell survival, differentiation, and migration. Previous reports have shown the importance of the Insulin-like Growth Factor II mRNA-Binding Protein (IGF2BP1/IMP1/ZBP1) in regulating RNA fate, including localization, transport, and translation. Here, we generated and characterized a knockout mouse to study RBP regulation. We report that IGF2BP1 is essential for proper brain development and neonatal survival. Specifically, these mice display disorganization in the developing neocortex, and further investigation revealed a loss of cortical marginal cell density at E17.5. We also investigated migratory cell populations in the IGF2BP1${}^{-/-}$ mice, using BrdU labeling, and detected fewer mitotically active cells in the cortical plate. Since RNA localization is important for cellular migration and directionality, we investigated the regulation of *β*-actin messenger RNA (mRNA), a well-characterized target with established roles in cell motility and development. To aid in our understanding of RBP and target mRNA regulation, we generated mice with endogenously labeled *β*-actin mRNA (IGF2BP1${}^{-/-}$; *β*-actin-MS2${}^{+/+}$). Using endogenously labeled *β*-actin transcripts, we report IGF2BP1${}^{-/-}$ neurons have increased transcription rates and total *β*-actin protein content. In addition, we found decreased transport and anchoring in knockout neurons. Overall, we present an important model for understanding RBP regulation of target mRNA.

## Significance

The insulin-like growth factor II mRNA-binding protein (IGF2BP1/IMP1/ZBP1) is an important RNA-binding protein (RBP) involved in regulating mRNA fate. Previous work on IGF2BP1 has served as an important model for understanding other RBPs. In this work, we present an IGF2BP1 knockout mouse model with endogenously labeled target mRNA (*β*-actin). This approach will expand our understanding of a critical RBP. In this study, we establish the essential role of IGF2BP1 in development and postnatal survival. In the absence of IGF2BP1, we also report endogenous *β*-actin mRNA transport and anchoring are significantly decreased at steady state. Additionally, we find that IGF2BP1 knockout neurons have increased actin protein content. Hence, IGF2BP1 is a major regulator of cell fate including transport, anchoring, and translation.

Local translation enables cells to respond quickly to local stimuli, supporting cellular processes such as fibroblast migration and neuronal synaptic plasticity (1, 2). RNA-binding proteins (RBPs) regulate local translation by trafficking messenger RNAs (mRNAs) to distal cytoplasmic regions. Local pools of mRNA provide a rapid source for protein synthesis, thereby allowing quick regulation of gene expression (3–5). Defects in local translation are implicated in physiologic processes like synaptic plasticity as well as diseases including amyotrophic lateral sclerosis, fragile X syndrome, and cancer (1, 6, 7).

Trafficking mRNAs requires high-affinity interactions between RBPs and their target mRNAs. The interaction between RBPs and mRNA is fundamental to regulating the mRNA life cycle (8). As such, defects in RBPs have been implicated in development including the insulin-like growth factor II mRNA-binding protein (IGF2BP1) and fragile X retardation protein (FMRP) (9, 10). RBPs interact with target mRNAs and other regulatory proteins (3, 11–13). These complex interactions form an RNA granule or a messenger ribonucleoprotein complex (mRNP) (4, 14). This mRNP complex can assemble or disassemble in response to local cues to facilitate local translation (3–5).

Much of what is understood about RBP regulation of mRNA transport, anchoring, and translation is derived from early studies of IGF2BP1 and its role in localizing *β*-actin mRNA. IGF2BP1 was identified and cloned from chicken and named the zipcode binding protein 1 (ZBP1) due to its association with an mRNA sequence important for mRNA localization (15, 16). Homologs were later discovered in *Xenopus* and *Drosophila* and named RBP-Vg1/Vera and dIMP, respectively (17). In contrast to chicken, frog, and flies, mammals encode a family of three IGF2BPs instead of one. In mammals, the homologs are referred to as IGF2BP1, IGF2BP2, and IGF2BP3, which correspond to their gene names, respectively. In this study, we will refer to the chicken homolog as ZBP1 and the mammalian homolog as IGF2BP1.

IGF2BP1 is necessary for cellular polarity and focal adhesion stability (18–22). In neurons, it is required for growth cone migration (23, 24). In addition to growth cone steering, IGF2BP1 also facilitates dendritic branching, filopodia, and synapse formation (23, 25–29). Hence, IGF2BP1 is a key component of regulatory complexes important for cellular behavior.

Dysregulation of IGF2BP1 has been implicated in development and cancer (7, 9, 30, 31). Unlike some RPBs, the spatial and temporal expression of IGF2BP1 is tightly regulated and largely limited to embryogenesis (30). The malignant function of IGF2BP1 appears when it is up-regulated [reviewed in (7)].

Prior work demonstrated IGF2BP1 has important roles in kidney and gut development but not in other organs (30). A window of temporal regulation of IGF2BP1 in the brain (peaking at E12.5 and decreasing by E17.5) has been reported by others (32); they did not detect abnormalities in the brain in their IGF2BP1 knockout mouse. Subsequent investigations showed that the model system was not a complete knockout but rather a hypomorphic mutant (31), possibly explaining the limited phenotypic changes. These mutant mice had a smaller cerebral cortex and decreased stem cell differentiation into neuronal and glial during E12.5 and E14.5 (33). In addition, these hypomorphic mutants exhibited impaired axon guidance (34), and haplosufficient mutants from this mouse model showed restricted axonal mRNA localization (35). Independent work showed that IGF2BP1 has important roles in cortical neurons and IGF2BP1-deficient mice display axonal defects in growth cone steering (24, 25). Molecular characterization of these phenotypes in IGF2BP1-deficient neurons further showed reduction in *β*-actin mRNA localization after stimulation. While this work provided important clarification on the molecular role of IGF2BP1, it did not characterize the gross effects of IGF2BP1 on the developing mouse.

IGF2BP1 has an important role in *β*-actin mRNA transport, dendritic anchoring, and local translation (15, 16, 20, 36, 37). Previous work identified and characterized the ZBP1-*β*-actin mRNP using structural and single-molecule imaging approaches (16, 38–41). Cocrystals of the ZBP1 domains with *β*-actin mRNA showed that ZBP1 binds a bipartite sequence, resulting in loop formation (39, 40). It is proposed that this loop sequesters the *β*-actin stop codon to aid in translation regulation in a ZBP1 concentration–dependent mechanism (39–41). The precise role IGF2BP1 plays in regulating translation is unclear, as there are multiple studies reporting positive and negative effects on *β*-actin translation. Studies from neurons have shown that reduction or loss of IGF2BP1 leads to lower levels of *β*-actin protein in distal dendrites and growth cones, yet higher overall actin protein content. This suggested that IGF2BP1 facilitates local translation (23, 27). In contrast, inhibition of translation by ZBP1 bound to the *β*-actin mRNP may prevent ribosomal subunits from binding and initiating translation of *β*-actin (42). Additional work using fluorescence correlation spectroscopy demonstrated the ZBP1–*β*-actin mRNA interaction anticorrelates with ribosome occupancy, supporting a role for translational repression (38). Further investigation into this regulation of translation revealed that phosphorylation of ZBP1 by Src kinase enhanced local *β*-actin translation (42–44).

In order to study the direct effects of IGF2BP1, a model RBP, on target mRNAs, we characterized a mouse model where *β*-actin mRNA was tagged with GFP. We investigated the phenotypic effects of null mice during development. The spatial and temporal regulation of IGF2BP1 makes it an excellent model to study how RBPs affect brain development and determine the direct effects they exhibit on target mRNAs. Our work complements previous IGF2BP1 model systems by examining the phenotypic changes in null mice and identifying direct effects on endogenous *β*-actin mRNA localization, transport, and anchoring. This mouse model will continue to serve as an important reagent for understanding how RBPs regulate target mRNAs.

## Results

### IGF2BP1${}^{-/-}$ Mice Exhibit Perinatal Lethality with Neocortex Disorganization.

**Fig. 1.**
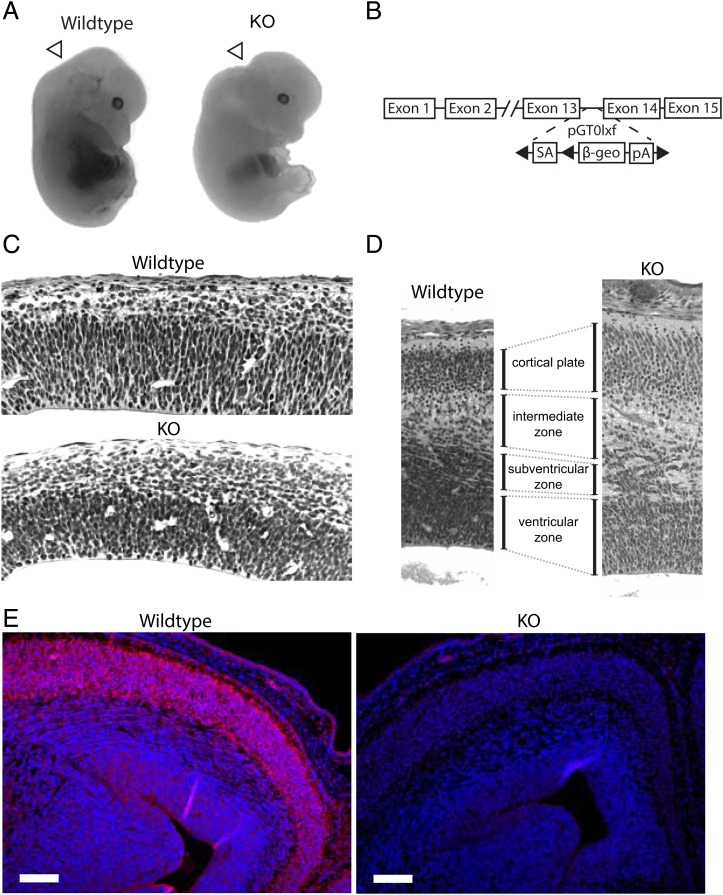
IGF2BP1 is required for proper neocortical organization. (*A*) Wildtype (WT) and knockout (KO) embryos pictured at E14.5. Gross deformity of posterior brain is visible at this stage, indicated by white arrows. (*B*) Schematic of gene-trap strategy used to generate IGF2BP1KO mice. A *β*-geo cassette was inserted between the 13th and 14th exon. (*C*–*E*) Coronal sections through the neocortex at (*C*) E14.5 and (*D*) E17.5 (20× magnification). Neuroepithelial cells in IGF2BP1 KO mice failed to orient in palisading fashion around lateral ventricles and, instead, displayed more rounded nuclei without apparent orientation. (*E*) Immunofluorescence against IGF2BP1 (magenta) at E17.5 stained highest for IGF2BP1 in the cortical plate in wild-type embryos. No expression was observed in the IGF2BP1${}^{-/-}$ mice. (Scale bar, 100 µ m.).

Elimination of IGF2BP1 expression was achieved through homologous recombination of a *β*-geo cassette into the 13th exon (Fig. 1*B*) [previously described in prior publications (20, 38, 40)]. Using this approach, we obtained an IGF2BP1-null variant distinct from previously reported mice (23, 30). We investigated whether the mice exhibited similar survival and developmental phenotypes.

Consistent with other IGF2BP1 knockout mice (30), we measured a dwarfism phenotype in all visceral organs (*SI Appendix*, TableS1). Similar to the IGF2BP1${}^{-/-}$ mouse (23), we found very few embryos survive after birth in the absence of IGF2BP1 (Table 1 and *SI Appendix*, Fig.S2*A*) (23). Surprisingly, we found no difference in the histopathology of visceral organs when comparing embryos at E14.5 and E17.5 (*SI Appendix*, Fig.S3).

**Table 1.** Survival statistics and distribution of genotypes of progeny from IGF2BP1${}^{+/-}$ x IGF2BP1${}^{+/-}$ crosses

Stage	WT	HET	KO
E14.5	8 (24%)	17 (52%)	7 (21%)
E17.5	3	8	6
P1	3	4	0
Total progeny	311 (41%)	444 (58%)	5 (0.6%)

Embryo and postnatal survival characterized in wildtype (WT), heterozygote (HET), and knockout (KO).

Gross inspection of the IGF2BP1 knockout mice compared to wild-type mice displayed deformities in the posterior brain at E14.5, suggesting development of the brain was affected (Fig. 1*A*). The deformities observed corresponded with previous descriptions of IGF2BP1’s expression, which is up-regulated at E12.5 (30) and persists throughout development. Based on these observations, we further characterized cellular changes in the developing brain. Midbrain coronal sections stained with hematoxylin and eosin (H&E) from E14.5 IGF2BP1${}^{+/+}$ and IGF2BP1${}^{-/-}$ littermates revealed an apparent neuroepithelial orientation defect in mice without IGF2BP1. Failure of cells to arrange in a palisading manner around the lateral ventricles indicated a cellular packing deficiency in IGF2BP1 null embryos (Fig. 1*C*). Since most neuronal differentiation and migration into the cortical layers occurs from E14.5 to birth, patterns of the cerebral cortex were assessed at E17.5. Coronal serial sections of E17.5 brains were taken from wild-type and IGF2BP1${}^{-/-}$ littermates and assessed with H&E staining for histopathological differences. Disorganization and deficient cellular packing were persistent in IGF2BP1 null brains at E17.5 (Fig. 1*D*). The intermediate zone was expanded with a poorly defined barrier to the subventricular zone in the IGF2BP1${}^{-/-}$ embryos. An indication that migration may be the source of this patterning defect came from the observation of a reduced cell density in the subcortical region in E17.5 IGF2BP1 KO embryos (*SI Appendix*, Fig. S2*B*). This suggested a reduced migration of newly differentiated interneurons into the cortical plate from the medial ganglionic eminence.

### IGF2BP1 Is Required for Neural Migration into the Cortical Plate.

**Fig. 2.**
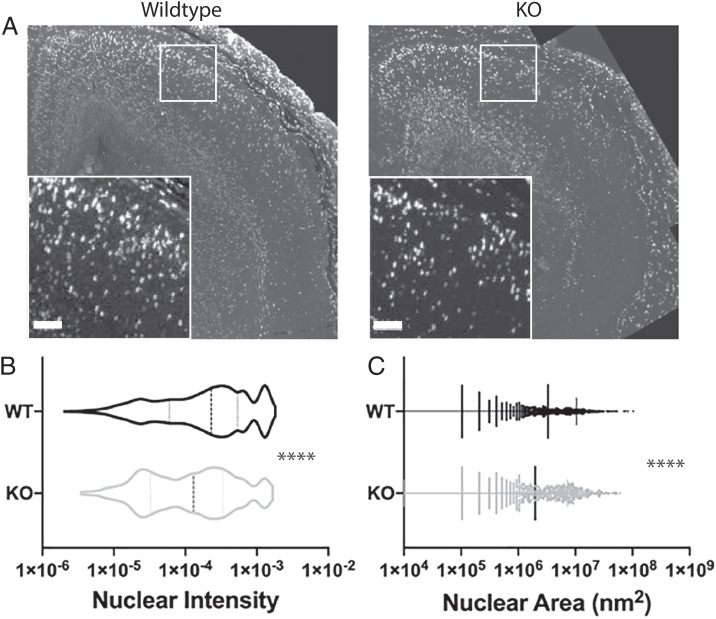
IGF2BP1${}^{-/-}$ embryos have reduced neural mitotic activity in the cortical plate. BrdU was administered by intraperitoneal injection at E14.5 and E15.5 into pregnant dams, which were killed 2 d later. (*A*) Immunofluorescent staining for mitotically active cells showed fewer BrdU-positive cells in the midbrain neocortex in the IGF2BP1 KO by coronal sections (10X magnification). Insert represents magnified area with a scale bar representing 25 µ m. (*B*) Quantification of BrdU intensity in wild-type and knockout embryos. Mean intensity was normalized by dividing by the area (square nanometers). (*C*) Area of BrdU staining in WT and KO. The total number of BrdU-positive nuclei detected were 2,036 for WT and 936 for KO. ****P < 0.0001, unpaired *t* test.

To test whether newly differentiated neurons had reduced migration into the cortical plate, BrdU was injected into pregnant mice by intraperitoneal injection at E14.5/15.5, and the mice were killed 2 d later. Littermates were compared directly for how many BrdU-positive cells were stained by immunofluorescence in the cortical plate. We observed a consistent reduction and inconsistent pattern of BrdU-positive cells in the cortical plate from IGF2BP1${}^{-/-}$ embryos (Fig. 2). Additionally, BrdU-positive nuclei were brighter and more numerous in the ventricular zone at E17.5 in IGF2BP1${}^{-/-}$ embryonic brains with a paucity of cells in the cortical plate (total number of BrdU-positive nuclei WT = 2,036, KO = 936). This could indicate an inability of neurons to radially migrate into the cortical plate, which correlates with previous data that show a deficiency in directed motility in KO cells (20).

### Loss of IGF2BP1 Results in Increased Cellular *β*-Actin mRNAs, but Dendritic *β*-Actin mRNAs Remain Unchanged in Distribution.

**Fig. 3.**
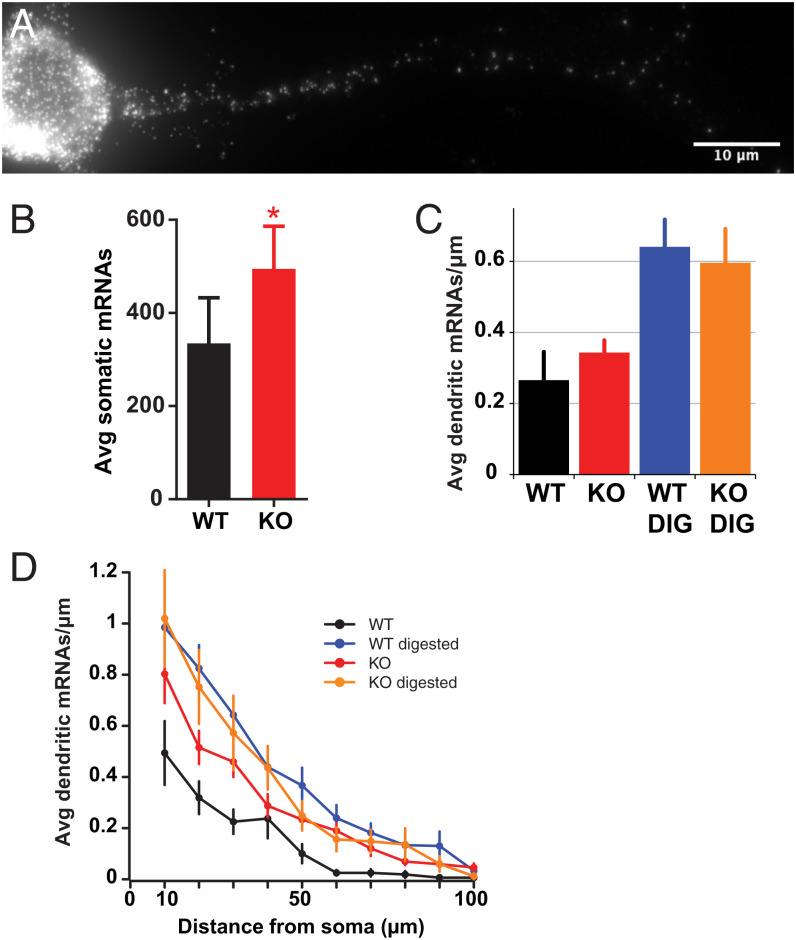
Average number of somatic *β*-actin mRNAs increases but dendritic distributions remain unchanged in the absence of IGF2BP1. (*A*) Representative smFISH of *β*-actin–MS2 stem loops (MBS) mRNA in IGF2BP1${}^{-/-}$ neurons. (Scale bar, 10 µ m.) (*B*–*D*) Total transcripts increase by 48% in KO smFISH quantified in wild-type (WT) and IGF2BP1 null (KO) neurons. (*B*) Average number of somatic mRNAs per neuron. *P=0.0171. (*C* and *D*) Protein digestion of mRNP granules used to reveal masked dendritic mRNAs. DIG represents protease digested samples. (*C*) Mean number of dendritic *β*-actin mRNAs per micrometer. Bars, SEM. (*D*) Average dendritic *β*-actin transcripts as a function of distance from soma; n= WT, 33; KO, 25; WT DIG, 16; KO DIG, 39.

To understand how IGF2BP1 regulates target mRNAs, we studied the effects of IGF2BP1 knockout on mRNA distribution, transport, and anchoring. We used single-molecule fluorescence in situ hybridization (smFISH) to detect *β*-actin mRNA distribution. We found increased transcription in the knockout neurons (*SI Appendix*, Fig. S1). In accordance with increased transcription, we found up-regulation in the average number of somatic *β*-actin transcripts (Fig. 3*B*). Surprisingly, we did not see any difference in the number of mRNAs present in dendrites. To understand whether mRNP granules were masking additional mRNAs, we performed protein digestion prior to fixing cells. After digestion, we found no statistical difference in the number of dendritic *β*-actin mRNAs, regardless of the distance from the soma (Fig. 3 *C* and *D*). While we found that *β*-actin mRNA distribution did not differ between wild type and knockout in the dendrites, we previously reported changes in other mRNAs present in dendrites. For example, spinophilin, a dendritic spine protein, had fewer mRNAs in the dendrites of IGF2BP1KO neurons (40), unlike *β*-actin mRNA. Knockout of *β*-actin is highly lethal and results in early mouse lethality (E9.5) (45). Given the essential role of this cytoplasmic actin isoform, it follows that there are likely redundant regulatory mechanisms to preserve *β*-actin content within the cell to avoid detrimental effects.

### *β*-Actin mRNPs in IGF2BP1${}^{-/-}$ Neurons Display Decreased Anchoring and Active Transport Compared to Wild Type.

**Fig. 4.**
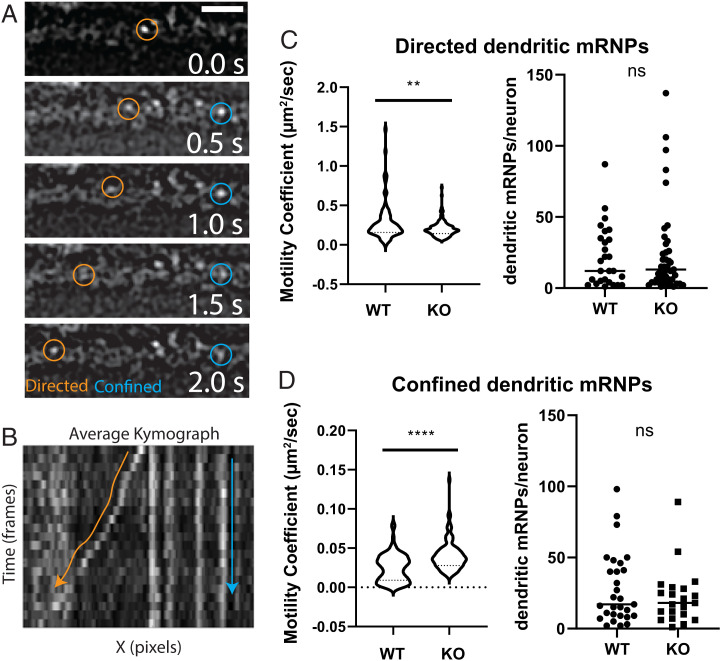
At steady state, dendritic *β*-actin mRNP transport and anchoring are decreased in knockout neurons. (*A*) Directed (orange) and confined (blue) mRNPs tracked along a dendrite. (Scale bar, 3 µ m.) (*B*) Kymograph of the movie from *A*. Estimates for the motility coefficients for wild-type and IGF2BP1${}^{-/-}$ neurons were determined using mean-square displacement. (*C*) (*Left*) The mean motility coefficient for directed mRNPs is lower in IGF2BP1${}^{-/-}$ neurons (number of mRNPs; WT = 87, KO = 137). (*D*) (*Left*) In contrast, the average motility coefficient of confined/anchored mRNPs is higher for knockout neurons (number of mRNPs; WT = 98; KO = 103). (*C* and *D*) (*Right*) The total number of directed mRNPs were calculated per neuron. There was not a statistical difference in the number of directed or confined mRNPs found in WT vs. KO (directed, *P* = 0.8495; confined, *P* = 0.3801). Neurons from three independent experiments were evaluated (number of neurons, WT = 32, KO = 48). ${}^{****}P<0.0001,{\;}^{**}P=0.0046$, ns = not significant, unpaired *t* test.

Next, we investigated whether mRNP dynamics were altered in the absence of IGF2BP1. Using live imaging, we tracked endogenously labeled *β*-actin mRNPs in wild-type and IGF2BP1${}^{-/-}$ dendrites at steady state. Individual *β*-actin mRNPs were categorized by their motion type using mean-square displacement analysis. We used directed and confined motion as quantitative approximates for active transport and anchoring states, respectively. We found alterations in the motility coefficients for both directed and confined motion in knockout neurons. Specifically, directed *β*-actin mRNPs displayed slower motility coefficients in IGF2BP1${}^{-/-}$, suggesting a defect in transport (Fig. 4*C*). In contrast, confined *β*-actin mRNPs had faster motility coefficients in the absence of IGF2BP1, suggesting an impairment in IGF2BP1-dependent anchoring (Fig. 4*D*). In addition, after quantifying the number of mRNPs per neuron in each motion, we did not find a change in the relative distributions. Thus, the changes in motility coefficients confirm IGF2BP1 is playing a role in active transport and anchoring of *β*-actin mRNPs within dendrites.

### IGF2BP1-Deficient Neurons Have Larger Cell Bodies and More *β*-Actin Protein Content.

**Fig. 5.**
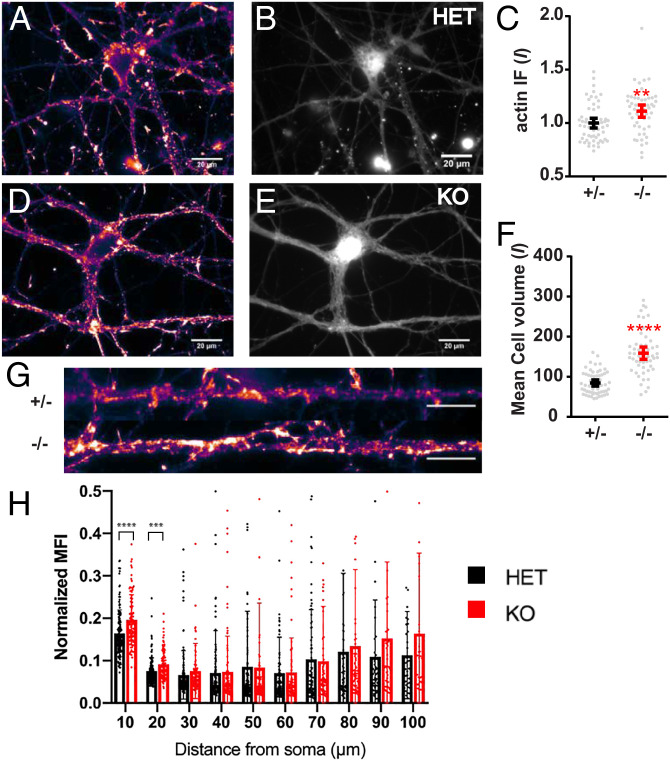
Loss of IGF2BP1 results in increased actin protein content and cell size. (*A* and *D*) *β*-actin protein immunofluorescence in cultured IGF2BP1${}^{+/-}$ (HET) and IGF2BP1${}^{-/-}$ (KO) neurons. (*B* and *E*) CellTracker fluorescence in IGF2BP1${}^{+/-}$ and IGF2BP1${}^{-/-}$ neurons to represent the volume of the neuron. (*C*) Quantification of *β*-actin immunofluorescence intensity in IGF2BP1${}^{+/-}$ and IGF2BP1${}^{-/-}$ neurons. Protein change between HET and KO is 11%. (*F*) Mean CellTracker fluorescence intensity in cultured neurons. (*G*) Example of straightened dendrites in HET and KO. (Scale bar, 10 µ m.) (*H*) Normalized mean fluorescence intensity (MFI) is calculated by dividing the mean fluorescent intensity by the area. Function of the distance from the soma is in micrometers. *N*, Het = 52 images, two coverslips; KO = 58 images, two coverslips. **P = 0.0013, ***P = 0.0002, ****P < 0.0001, unpaired *t* test.

Since IGF2BP1 plays an important role in regulating translation of *β*-actin, we sought to evaluate whether the protein concentration of *β*-actin was altered in the absence of IGF2BP1. We probed against *β*-actin protein with immunofluorescence and used a cell volume marker to determine cell size (Fig. 5). When comparing the heterozygous to the homozygous IGF2BP1 neurons, complete loss of IGF2BP1 resulted in higher cell volume and immunofluorescence intensity (Fig. 5 *E* and *F*). In comparison to the up-regulation of *β*-actin transcription in knockout neurons (transcript change = 48% increase), we only found an 11% increase in actin protein content in knockout neurons.

## Discussion

In this investigation, we confirm that IGF2BP1 is essential for mouse survival, consistent with previous reports (23, 30, 42). The critical role of IGF2BP1 during development is conserved across multiple organisms. In *Drosophila*, loss of the ortholog, dIMP, resulted in increased fly lethality during development, especially in the late pupal and early adult pharate stages (46). Interestingly, this corresponds to similar lethality (i.e., late development and postnatal stages) that we find in the IGF2BP1 null mouse. The role of IGF2BP1 ortholog in neurogenesis is also conserved, as neural defects have been reported in multiple model organisms including *Xenopus*, *Drosophila*, and mice (17, 33, 46). In zebrafish, knockout affects axonal outgrowth (47).

This characterization of the gene-trap mouse extends previous studies to understand the functional relevance of IGF2BP1 in brain development. We find that this RBP is critical to proper corticogenesis as seen by neuronal patterning. The aberrations in neuronal patterning and mitotic activity deficits are most striking at embryonic day 14.5, correlating with a brief period with high expression. These observations underline the importance of IGF2BP1 in development of the brain, based on its tightly controlled expression pattern, which were previously shown for hypomorphic mutants (33).

We also verified the importance of IGF2BP1 in regulating endogenous target mRNA. Using *β*-actin mRNA as a model target, we followed the effects on mRNA distribution, transport, and anchoring. While null mice have increased somatic mRNAs, the number of dendritic mRNAs remains unchanged, suggesting that mRNA distribution is not affected by the loss of this RBP. Previous reports have shown *β*-actin mRNA distribution was reduced after transient loss of IGF2BP1 (27). Yet, we did not find changes in dendritic distribution after chronic loss. Chronic loss may allow the redundant mechanisms to compensate for *β*-actin perturbations, in comparison to transient loss. While these compensatory mechanisms may be sufficient to sustain *β*-actin levels at steady state, neuronal stimulation has been shown to increase IGF2BP1 distribution in dendrites (23, 26, 27). Thus, stimulation conditions may enable the effect that IGF2BP1 has on mRNA distribution. In addition, IGF2BP1 may have differential effects on target mRNAs. In contrast to *β*-actin mRNA, fewer spinophillin mRNAs are distributed within dendrites in IGF2BP1 null neurons (40). This suggests that the regulatory role IGF2BP1 has in mRNA distribution and localization may be more robust for nonessential genes.

To understand the role of IGF2BP1 in transport and anchoring, we characterized changes in the motility of dendritic mRNPs. We found that transport mRNPs were less motile in knockout dendrites. However, we did not find a statistical difference in the number of transported mRNPs in IGF2BP1${}^{-/-}$ dendrites. Therefore, active transport still occurred in the absence of the RBP, but it occurred, on average, at a slower rate. Similarly, we also found changes in anchored mRNPs. Anchored mRNPs were, on average, more motile in knockout dendrites, suggesting the loss of an anchoring component. This is consistent with IGF2BP1 acting as an anchor in dendrites (23, 37).

The anchoring behavior seen in this study is complementary to previous work. In prior work, a neuron was stimulated at a dendritic spine using glutamate uncaging. Within minutes, *β*-actin mRNA localized to the base of the stimulated spine. Once localized, the mRNA was anchored at the site for several hours. If there were no further stimulations, the mRNA resumed directed and diffusive movements. The anchoring required IGF2BP1, since localized mRNA was reduced in knockout cells (37). In contrast to previous work, we evaluated all *β*-actin MBS-tagged mRNAs within dendrites. Most of the mRNAs exhibited nonmotile behavior; localized and translating mRNAs represented a minority of these mRNAs (37, 48). While we did find small differences in the anchored population between wild type and knockout, the analysis was skewed by the large number of nonmotile mRNAs which lack localization and translation. Therefore, global measurements of mRNA kinetics may underestimate changes occurring within subpopulations. Overall, small changes in anchored motion may still represent important changes to critical subpopulations including localized and translating mRNAs.

To further understand IGF2BP1’s role in regulating *β*-actin, we also investigated whether loss of IGF2BP1 affected the total *β*-actin protein content. Despite robust transcriptional up-regulation of *β*-actin in knockouts, we anticipated small changes in protein levels, because *β*-actin is an essential cytoskeletal protein. Previous studies have indicated that actin protein levels are maintained even when *β*-actin mRNA is overexpressed (49). Taken together, maintaining cytoskeletal proteostasis is critical to cellular life, and there are likely multiple mechanisms that ensure the protein levels are maintained (50).

In summary, IGF2BP1 has served as an important model RBP. Our work further establishes it as an essential gene in postnatal survival. In addition, we demonstrated this RBP has important effects on mRNA fate including transport and anchoring. Yet, it is unclear, in the absence of IGF2BP1, whether compensation by other structurally similar RBPs occurs. Further investigation is needed to address how RBPs may act combinatorially to regulate *β*-actin mRNA distribution and local translation.

## Materials and Methods

### Animals.

IGF2BP1 null mice were generated through homologous recombination of the *β*-geo cassette between the 13th and 14th exons of IGF2BP1. Identification of the insertion site by 5′RACE revealed the cassette recombined into the 13th of 15 exons. The 129P2/OlaHsd embryonic stem cells containing XL009 gene traps were injected into C57BL/6 blastocysts, and progeny were crossed into a C57BL/6 background (BayGenomics). IGF2BP1${}^{+/-}$ mice were bred with MBS mice (51) to produce IGF2BP1${}^{+/-}$MBS${}^{+/+}$ double-transgenic animals. We confirmed complete knockout of IGF2BP1 by PCR of the *β*-geo cassette from the 13th to 14th exon (20). To ensure the absence of a functional fusion protein, Western blot was performed using cells derived from IGF2BP1${}^{-/-}$ embryos against multiple antibodies toward the n-terminus. We did not identify IGF2BP1 protein in IGF2BP1${}^{-/-}$ embryos (20, 38). Additionally, qPCR toward the 5′ region revealed significantly reduced mRNA quantities in IGF2BP1${}^{-/-}$ cells when compared to IGF2BP1${}^{+/+}$ cells. To isolate embryos for this study, IGF2BP1${}^{+/-}$ X IGF2BP1${}^{+/-}$ matings were set up in a 2-d window.

### Histopathological Staining and Sectioning.

Embryos were isolated at the desired developmental window and immediately submerged in cold 4% paraformaldehyde. After 24 h at 4 ^∘^C, embryos were transferred to 70% ethanol (ETOH) and processed for histological slicing after paraffin embedding. All visceral organs were isolated from E14.5 and E17.5 embryos and stained with H&E to assess for differences between wild-type and IGF2BP1${}^{-/-}$ embryos. Brains were processed by serial sections to identify differences between the two genotypes. Once the midbrain neocortex histopathology was identified, further embryos were processed in this area of the brain with coronal sections. Slides were deparaffinized with heat and xylene to melt the wax. Serial dilutions from 95% to water were used to rehydrate the sections.

### Brain Immunofluorescent Staining.

Epitope retrieval was performed by boiling samples in 10 mM sodium citrate for 20 min, then washing with PBS. Samples were then neutralized with 0.1 M sodium borate buffer (pH 8.5) and washed twice with 0.1% triton X-100. Samples were then blocked with appropriate blocking solutions (either normal goat serum or donkey serum, depending on secondary antibody) and stained by traditional immunofluorescence protocols. Primary antibodies used include a previously reported polyclonal rabbit anti-IGF2BP1 antibody (52) and rat anti-BrdU (Abeam, Ab6326). Secondary antibodies used include a donkey anti-Rat Cy5 and Cy3 conjugated donkey anti-rabbit (both from Jackson ImmunoResearch).

### BrdU Labeling and Quantification.

BrdU (6 mg) was administered by intraperitoneal injection at E14.5 and E l 5.5. Mothers were killed at El7.5, and embryos were isolated for immunohistochemistry processing as described above. For BrdU staining samples, the immunofluorescent procedure above was followed. Images were quantitated in FIJI. First, a mask of the neocortex was created. Then an intensity threshold (127 to 255) was set to identify BrdU-positive nuclei within the mask. Pixels were converted into nanometers (1 pixel = 322.5 nm).

### Neuron Cultures.

IGF2BP1${}^{+/-}\beta$-actin–MS2${}^{+/+}$ and IGF2BP1${}^{+/-}\beta$-actin–MS2${}^{+/+}$ were crossed and genotyped. Cultures were then used for live imaging, single-molecule FISH, and immunofluorescence experiments. Live imaging of *β*-actin mRNA in IGF2BP1${}^{+/+}$MBS${}^{+/+}$ and IGF2BP1${}^{-/-}$MBS${}^{+/+}$ neurons was performed as described in refs. 37, 38, and 40. In brief, murine cultures were isolated from E18 hippocampi (53). Neuronal cultures were performed at 21 d to 28 d in vitro.

### FISH and Digestion.

Single-molecule labeling of endogenous *β*-actin–MBS mRNAs and digestion to unmask mRNAs were performed as per ref. 3.

### Fixed Cell Image Acquisition and Analysis.

The smFISH images were acquired with an Olympus BX-61 microscope. Microscope specifications and quantitative image analysis are described in refs. 3 and 40.

### Single-Particle Kinetic Analysis.

Live images were analyzed using Diatrack as previously described (54, 55). Published and custom scripts were used to analyze and discriminate motion types and calculate motility coefficients (56–58).

## Supplementary Material

Supplementary File

## Data Availability

Scripts are available on GitHub (https://github.com/leti332/dendritic-mRNA-analysis) (59). Imaging data is available upon request from authors. All other study data are included in the article and/or *SI Appendix*.
