# Supplementary material for: Tagged actin mRNA dysregulation in IGF2BP1−/− mice
Source: Proc Natl Acad Sci U S A. 2022 Sep 6;119(37):e2208465119. doi: 10.1073/pnas.2208465119 (PMC9477413; doi:10.1073/pnas.2208465119)
Supplement: Supplementary File [file pnas.2208465119.sapp.pdf]

Supplementary Information for

Tagged Actin mRNA Dysregulation in IGF2BP1$^{-/-}$ mice

Leti Núñez, Adina Buxbaum, Zachary B. Katz, Melissa Lopez-Jones, Chiso Nwokafor, Kevin Czaplinski, Feng Pan, Jason Rosenberg, Hannah Monday, Robert H. Singer

Robert H. Singer. To whom correspondence should be addressed. E-mail: Robert.Singer@einsteinmed.edu

This PDF file includes:

Figs. S1 to S3
Table S1

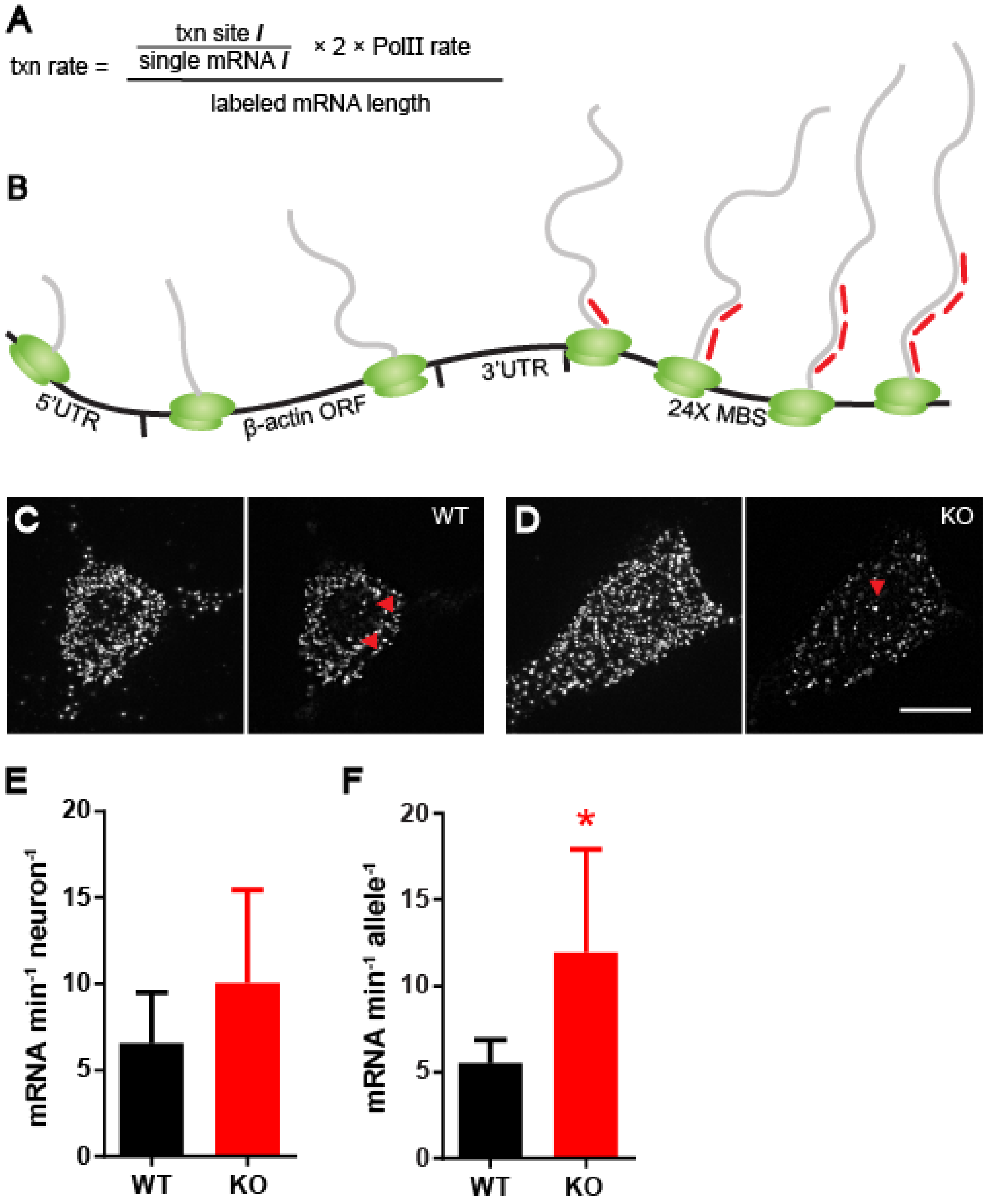

Fig. S1. IGF2BP1 KO neurons have enhanced β-actin mRNA production rates.(A) Equation for calculating rates of β-actin mRNA transcription. mRNAs at transcription sites is multiplied by the transcription elongation rate of RNA polymerase, normalized for the length of the mRNA (See Materials and methods). (B) Illustration of FISH labeling of nascent mRNAs at transcription sites. Green, PolII; Grey, nascent mRNA; Red, single MBS FISH probes. (C) Image of MBS FISH of WT neurons; Maximum projection of 41 planes (left); Single image plane (right). (D) Image of MBS FISH of IGF2BP1 KO neuron; Maximum projection (left); single plane (right). Red arrow heads specify transcription sites labeled by MBS FISH probes. (E) Histogram of transcription rates of β-actin mRNAs synthesized per minute per cell in WT and IGF2BP1 KO neurons calculated by the method described in (A). (F) Mean mRNA produced per minute in each neuron, same cells as (E). (G) Mean mRNA produced per minute per transcription site (allele). n=WT, 17; KO, 19 neurons. Bars, 95% CI; I, Intensity; MBS, MS2 binding sites.

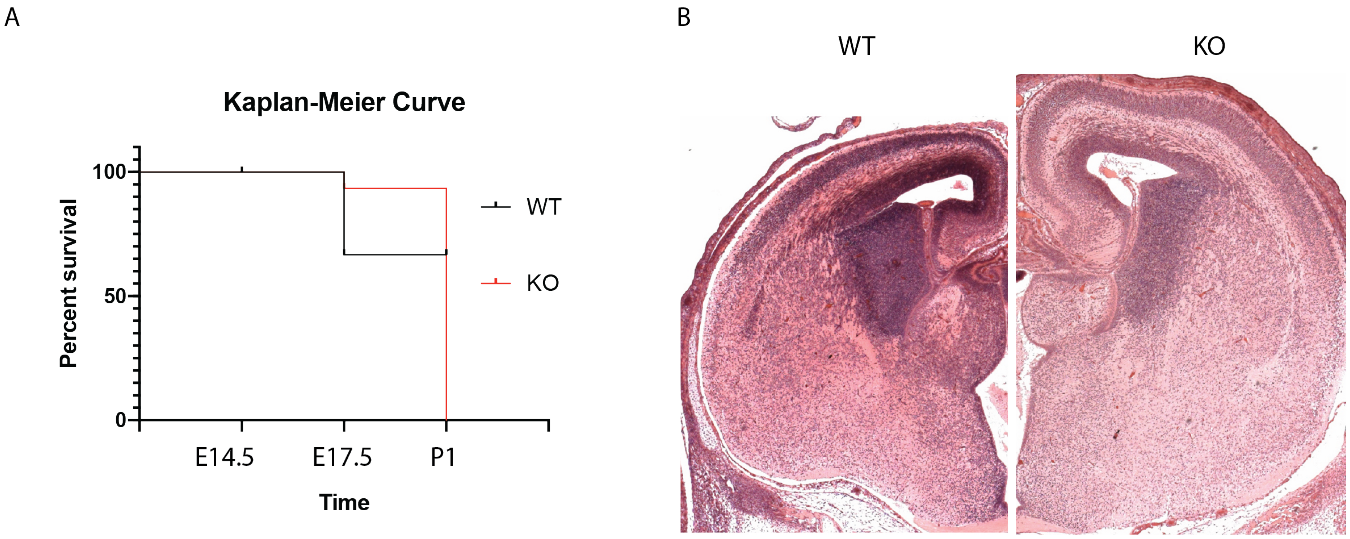

Fig. S2. IGF2BP1-/- mice exhibit perinatal lethality with reduced cell density in subcortical region (A) Kaplan-Meier Curve showing percent survival of wildtype versus IGF2BP1KO pups during development (E14.5 and E17.5) to birth (P1). (B) Cortex from wildtype and IGF2BP1 knockout mice with reduced subcortical cell density

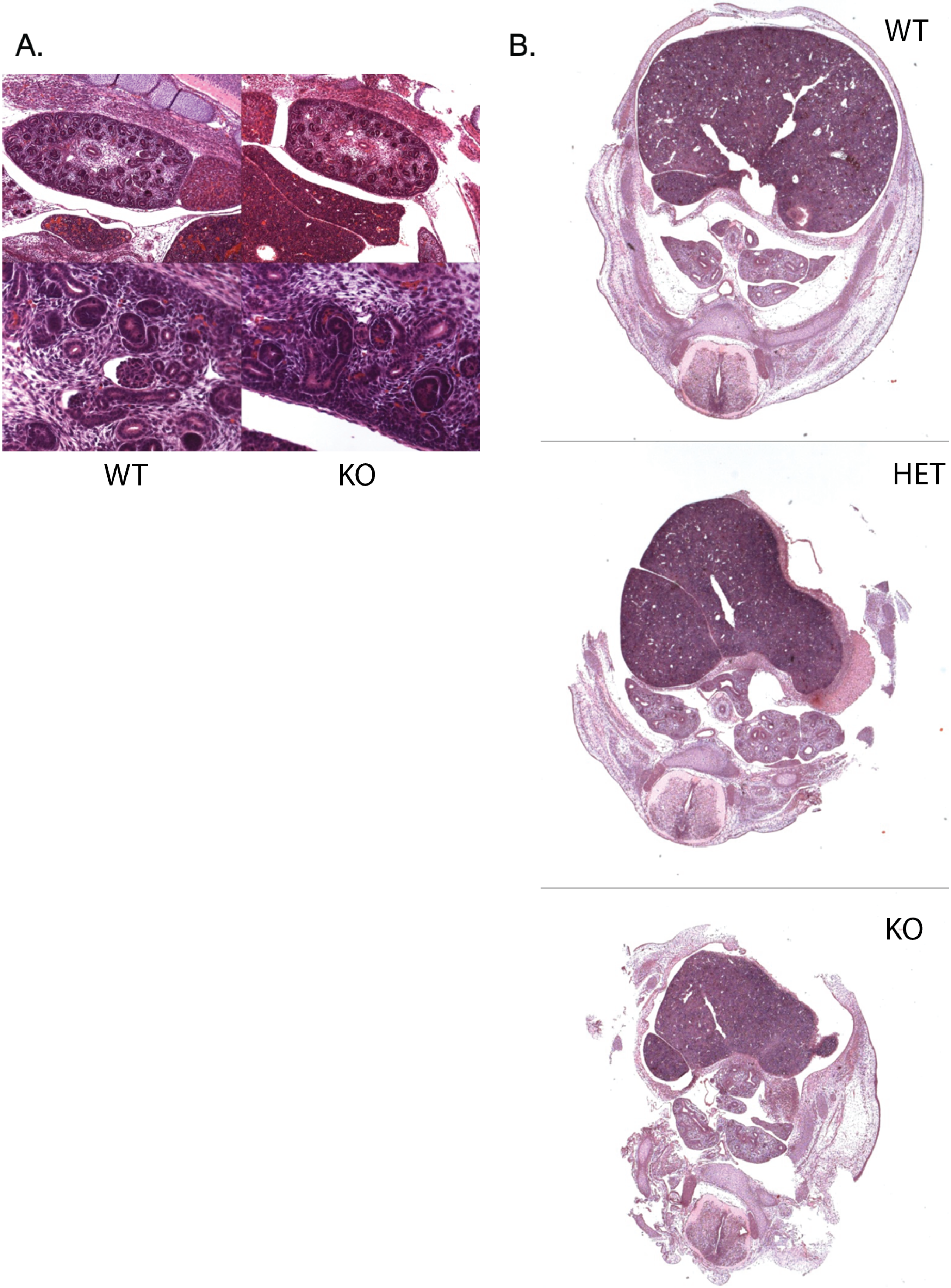

Fig. S3. Histopathological characterization of visceral organs. (A) Kidney (B) Liver

Table S1. IGF2BP1KO embryos (E18.5) have dwarfism phenotype of organs.

	Liver	Intestine	Heart	Lungs
WT	53 mg	20 mg	14 mg	24 mg
HET	38 mg	21 mg	16 mg	19 mg
KO	30 mg	15 mg	11 mg	14 mg
